# The cytokine milieu of bullous pemphigoid: Current and novel therapeutic targets

**DOI:** 10.3389/fmed.2023.1128154

**Published:** 2023-02-06

**Authors:** Roberto Maglie, Farzan Solimani, Dario Didona, Carlo Pipitò, Emiliano Antiga, Giovanni Di Zenzo

**Affiliations:** ^1^Section of Dermatology, Department of Health Sciences, University of Florence, Florence, Italy; ^2^Department of Dermatology, Venereology and Allergology, Charité – Universitätsmedizin Berlin, Corporate Member of Freie Universität Berlin, Humboldt-Universität zu Berlin, Berlin Institute of Health, Berlin, Germany; ^3^BIH Charité Clinician Scientist Program, Berlin Institute of Health at Charité – Universitätsmedizin Berlin, BIH Biomedical Innovation Academy, Berlin, Germany; ^4^Department of Dermatology and Allergology, Philipps University, Marburg, Germany; ^5^Laboratory of Molecular and Cell Biology, Istituto Dermopatico dell’Immacolata (IDI)-IRCCS, Rome, Italy

**Keywords:** cytokine, bullous pemphigoid, target therapy, interleukin, Th2, lymphocyte, eosinophil, neutrophil

## Abstract

Bullous pemphigoid (BP) is the most common autoimmune bullous disease, characterized by severe pruritus and skin blistering. The loss of tolerance against Collagen XVII, also referred to as BP180, is the main pathogenic event of BP, leading to production of IgG autoantibodies which mainly target the juxtamembranous extracellular non-collagenous 16th A (NC16A) domain of BP180. A complex inflammatory network is activated upon autoantibody binding to the basement membrane zone; this inflammatory loop involves the complement cascade and the release of several inflammatory cytokines, chemokines and proteases from keratinocytes, lymphocytes, mast cells and granulocytes. Collectively, these events disrupt the integrity of the dermal-epidermal junction, leading to subepidermal blistering. Recent advances have led to identify novel therapeutic targets for BP, whose management is mainly based on the long-term use of topical and systemic corticosteroids. As an example, targeting type-2 T-helper cell-associated cytokines, such as Interleukin-4 and interleukin-13 has shown meaningful clinical efficacy in case series and studies; targeting IL-17 and IL-23 has also been tried, owing to an important role of these cytokines in the chronic maintenance phase of BP. In this review article, we discuss the complex cytokine milieu that characterized BP inflammation, highlighting molecules, which are currently investigated as present and future therapeutic targets for this life-threatening disease.

## Introduction

Bullous Pemphigoid (BP) represents the most common autoimmune bullous disorder and prevalently occurs in the elderly (1). The disease is characterized by circulating IgG autoantibodies which mainly target the non-collagenous (NC)16A domain of Collagen XVII, also referred to as BP180, a main component of the hemidesmosomes, which maintains the integrity of the dermal-epidermal junction (DEJ) (2). Patients with BP also develop antibodies against BP230, a cytoplasmic protein of the hemidesmosomal plaque that cross-link BP180 to keratin (K) 5 and K14 (3, 4).

Bullous Pemphigoid encompasses a heterogeneous spectrum of manifestations. The classic type is characterized by diffuse tense blisters arising on a background of erythematous-edematous skin (5). Pruritus is always present and, in some patients, may precede for years the appearance of manifest lesions (6). Further, several non-bullous forms have been described (7).

Over the recent years, the incidence of BP is raising significantly (2, 8). This phenomenon is partly explained by an increasing aging population in western countries, and easier access to serological diagnostic kits. Epidemiologic studies showed that BP is associated to some neurological disorders (9–11), particularly dementia and Parkinson, drugs, such as dipeptidyl peptidase IV inhibitors (DPP4i) and PD-1 inhibitors (12, 13) and malignancies (14, 15). The pandemic showed that BP might occur after either SARS-CoV-2 infection or related vaccines (16, 17).

Pathogenically, IgG binding to either BP180 or BP230 activates a cascade of inflammatory mediators resulting in the loss of dermal-epidermal adhesion (18). The increasing knowledge of this complex inflammatory cascade is pivotal for developing new therapeutic strategies for the disease, as its therapeutic management is still largely based on long-term immunosuppressive treatments (10). Indeed, the purpose of this review is to provide a concise overview of the cytokine milieu of BP, with a special focus on molecules currently under-investigation as potential therapeutic targets.

## Cytokine regulation of humoral immunity in bullous pemphigoid

### Loss of tolerance against bullous pemphigoid antigens is associated with a prevalently Th2-type skewed immune response

**FIGURE 1:**
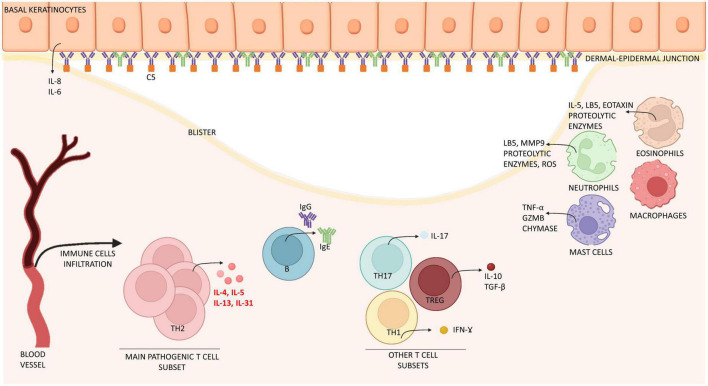
Schematic representation of immune cells and molecules involved in the pathogenesis of bullous pemphigoid. Bullous pemphigoid is determined by IgG, IgE attaching BP180 located in the dermal-epidermal junction. Epidermal cells react by releasing interleukin (IL) 6 and 8. Eventually, this process leads to the recruitment of immune cells (mast cells, macrophages, and eosinophils) which infiltrate the skin and release inflammatory interleukins (IL) and proteolytic enzymes. T cells contribute to this inflammatory process by releasing interleukins at both peripheral (blood) and lesional (skin) level. Especially IL-4, IL-13, IL-31 are crucially involved in B cell proliferation, antibody production and Ig-class switching, itch and eosinophils activation, while IL-17 support neutrophil recruitment. Together, immune cells induce expression of chemokines, thus increasing skin infiltration. The result of this process is the formation of erythematous urticarial plaques and, later, dermal-epidermal splitting causative of blistering. IL, interleukin; Ig, immunoglobulin; Th, T helper; TGF-β, tumor growth factor β; IFN-ɣ, interferon ɣ; GZMB, granzyme B; MMP9, matrix-metallopeptidase 9; LB5, leukotriene B 5; ROS, reactive oxygen species; C5, complement component 5.

BP180-NC16A-reactive CD4+ T cells play a pivotal role in the pathogenesis of BP (19). Although Th1 and Th2 mixed profiles were considered in the past as the main mediators of the immune response in BP (20), BP is currently regarded as a prevalently Th2-cell skewed disease (Figure 1). Pickford et al. demonstrated strong IL-4 and IgE responses in peripheral blood mononuclear cell isolated from BP patients when exposed to NC16A peptides (21). A recent study on Chinese population identified two NC16A peptides that were associated with the induction of a Th2-type immune activation in BP. Specifically, the authors demonstrated that Th2 cell activation in BP occurred in an human leukocyte antigen-DR (HLA-DR) restricted fashion. IL-4 production by activated Th2 cells was associated with B-cell activation and autoantibody production (22). Th2 cytokines IL-4, IL-13, and IL-31, which play also a crucial role in eosinophil chemoattraction, maturation and activity, and induction of pruritus, have been shown to increase both in the peripheral blood and skin lesions of BP patients (23, 24) (Figure 1 and Table 1). Here, chemokines such as eotaxin and MCP-4, whose levels increase in the blister fluid, support the chemotaxis of Th2 cells (25), supporting a positive feedback loop between activated Th2 cells and eosinophils.

**TABLE 1:** Overview of immunological players involved in the pathogenesis of bullous pemphigoid according to findings from serological, blister fluid, skin samples, and mice models analysis as well as their current clinical relevance based on ongoing and terminated clinical trials (published or registered at www.clinicaltrials.gov, accessed on 15 December 2022).

	Experimental evidence					Therapeutics		
**Molecule**	**Sera^1^**	**Blister fluid^2^**	**Skin^3^**	**Pathogenecity in mice^4^**	**Correlation to disease activity^5^**	**Targeted drug**	**Phase**	**NCT**
C5/LTB_4_	↑	↑	NN	+	NN	Avdoralimab Nomacopan	II III	NCT04563923 NCT05061771
Eotaxin-1	↑	↑	↑	NN	+	Bertilimumab	II	NCT02226146
IgE	↑	↑	NN	+	+	Omalizumab Ligelizumab	IV II	NCT00472030 NCT01688882
IL-4 IL-13	↑ ↑	↑ NN	↑ ↑	NN NN	NN +	Dupilumab	II/III	NCT04206553
IL-5	↑	↑	↑	NN	+	Mepolizumab Benralizumab	II III	NCT01705795 NCT04612790
IL-8	↑	↑	NN	+	+	DF2156A	II	NCT01571895
IL-17	↑	↑	↑	+	NN	Ixekizumab	II	NCT03099538
IL-23 (IL-12)	↑ NN	↑ NN	NN NN	NN NN	NN NN	Tildrakizumab Ustekinumab	I II	NCT04465292 NCT04117932

^1^Interleukin (IL) or other molecule levels in serum of patients. ↑ Indicates increased levels relative to control; NN not studied, or data are uncertain. ^2^IL or other molecule levels in blister fluid of patients. ↑ Indicates increased levels relative to control; NN not studied, or data are uncertain. ^3^IL or other molecule levels in affected skin of patients, detected by Immunohistochemistry/qPCR. + Indicates increased levels relative to control; NN not studied, or data are uncertain. ^4^Proven pathogenetic role in mice model. + Indicates confirmed pathogenetic role in mice model; NN not studied, or data are uncertain. ^5^Correlation with disease activity and/or severity in patients. + Indicates presence of correlation; NN not studied, or data are uncertain.

### B-cell subsets in the pathogenesis of bullous pemphigoid

B cell landscape of BP have been only poorly analyzed. Clinical evidence shows that B cells are pathogenically relevant, and their depletion with rituximab (RTX) is a viable therapeutic option, although less effective compared to pemphigus (26–31). This different qualitative effect exerted by RTX could be due to the persistency of IgE autoantibodies, which are still present also after RTX-treatment, and/or persistency of CD20- plasma cells (32). B-cells secreting autoantibodies were found also in BP lesions (33). Their trafficking into the skin seems to depend on the CXCR4/CXCL12 axis rather than cutaneous lymphocyte-associated antigen (CLA) expression, which is poorly expressed by B cells (34). Furthermore, CXCL12 activates C-Myc, which promotes B-cell differentiation into antibody-secreting cells and facilitate autoantibody production, and disruption of this axis results in altered antibody production *in vitro* (33).

B regulatory cells function in BP appears to be impaired. B regulatory cells are increased in the circulation of BP patients but show an inflammatory, rather than regulatory, phenotype secreting IFN-ɣ, IL-4, and TNF-α instead of IL-10 (35). Different studies reported that serum levels of B-cell activating factor (BAFF), a protein which regulates and stimulates B cell differentiation, is up-regulated in BP (36, 37).

### T follicular helper cells and altered T regulatory cells function support antibody production by autoreactive B-cells

T follicular helper (TFH) cells are a subset of T cells, characterized by the expression of CXCR5 and the capacity to migrate into germinal centers. TFH play a relevant role in autoimmune disorders by stimulating IgG switching and antibody production by activated B-cells (38). Inhibition of TFH cells represents a novel therapeutic approach in autoimmune diseases (39, 40). The signature cytokine of these cells is IL-21, which stimulate both TFH and B cell proliferation (41). Both TFH and IL-21 are increased in BP, positively correlating with anti-BP180 antibody levels; concordantly, treatment response in BP is accompanied by a decrease in TFH/IL-21 levels (42). Finally, absence of TFH cells or inhibition of IL-21 decreases autoantibody production by B-cells in *in vitro* T/B cell co-culture (42). In this context, also CXCL13, a chemokine which stimulate the migration of TFH cells is expressed in BP patients in both serum and skin (43).

T regulatory (T reg) cells are a crucial T cell subset in the pathogenesis of BP (Figure 1). Scurfy mice and patients affected by immune dysregulation, polyendocrinopathy, enteropathy, X-linked syndrome have been reported to spontaneously generate antibodies against BP180 and BP230, suggesting that altered Treg function increases the risk of developing BP (44). In BP, Treg cells increase in lesional skin (45–47), while IL-10 serum levels are up-regulated during disease remission (45). These observational data question whether skin infiltrating T regs show undiminished suppression capacity in BP.

## Interleukin-17/23 signaling in the pathogenesis of bullous pemphigoid

IL-17/IL-23 axis has a supportive role in the pathogenesis of BP. In a IL17^–/–^ mice model, passive transfer of anti-BP180 antibodies led to reduced skin inflammation, whereas in wild-type mice levels of IL-17 production following anti-BP180 IgG passive transfer correlated to disease severity (48). Collectively, this findings suggest that IL-17 is pathogenically relevant in murine BP. In human BP IL-17 levels increase in lesional skin and blister fluid (49) and its production is mainly sustained by neutrophils (Figure 1). In the peripheral blood of BP patients, CD3+ lymphocytes appear to be the main source of IL-17 (24). Interestingly, longitudinal measurement of IL-17 and IL-23 serum concentration was found to predict relapse in BP patients, as relapsing patients were shown to have persisting increased levels of serum IL-17 as well as increasing serum concentration of IL-23 during the first month of treatment (50). The IL-17/IL23 axis promotes various pathological processes, including DNA extracellular trap formation (51), stimulation of IL-1β production in macrophages and production of matrix-metalloprotease (MMP)-9 and neutrophil elastase, enzymes involved in blister formation (52, 53).

Interestingly, only a subset of BP patients shows up-regulation of IL-17 at the baseline, without correlation with disease severity, suggesting that not all the patients could effectively benefit from therapeutic targeting of the IL-17/IL23 axis (30–32). Interestingly, targeting IL17/IL23 demonstrated efficacy in patients with coexisting BP and psoriasis, a prototype of IL17/IL23 driven disease (33, 54–57). Results from ongoing clinical trials are thereby necessary to understand the impact of this treatment approach also in BP patients without coexisting psoriasis (58). Inhibition of janus kinase may worth being investigated in BP, since this could allow simultaneous targeting of both IL-4 and IL-23 (59–62).

## Complement-dependent and independent mechanisms contribute to blistering in bullous pemphigoid

Complement activation plays a pivotal role in the pathogenesis of BP (63, 64). This assumption is supported by several evidences: (i) the complement component C3 is disposed in a linear fashion along the DEJ in perilesional BP skin, and is even stronger than IgG, or sometimes found in the absence of IgG, in direct immunofluorescence (65–68); (ii) the capacity of autoantibodies to activate complement *ex vivo* correlates with disease activity and levels of autoantibodies in BP patients (69); (iii) milder clinical phenotype of BP230-type seems to correlate with weaker complement deposition at DEJ (70) (iv) genetic deficiency and/or pharmacological depletion of various complement components reduce pathogenicity of anti-BP180 IgG and dampen skin inflammation in experimental mouse models of BP (71, 72).

In BP patients, the classical pathway activation, which occurs following antibody/antigen binding, is the major pathway (73). *In vitro* and *in vivo* evidence suggests that blockage of C1q prevents both complement activation and skin blistering. Likewise, genetic absence of C4 in experimental BP mouse models is enough to abolish mast cell (MC) degranulation and attraction of neutrophils (71) (Figure 1). The alternative pathway of complement activation plays a supportive role in the pathogenesis of BP (71). Accordingly, passive transfer of anti-BP180 antibodies in mice with genetic deficiency of alternative pathway component FB developed a delayed and mild disease phenotype (71).

The activation of complement generates the attachment of component C3 and release of anaphylatoxins C3a and C5a, of which C5a can mediate polymorphonuclear leukocytes chemotaxis and both C3a and C5a can mediate MC degranulation (74). Of relevance in the generation of experimental BP in mice is the interaction between C5a and C5aR1 (75). C5aR2 conversely plays an anti-inflammatory and protective role in BP (76, 77). C5a receptor 1 (C5aR1) on MC was shown to be critical for the formation of skin lesions (74). Further, C5a/C5aR1 interaction on the surface of neutrophils activates the release of LTB4 *via* 5-lipoxygenase (78). Collectively, these molecules are indispensable to an efficient recruitment of neutrophils into the interstitial skin tissue (79, 80) (Figure 1 and Table 1).

A last consequence of complement activation in BP is the formation of the terminal membrane attack complex, which exerts direct cytotoxic effects in the epidermal basal cells.

It is worth noting that loss of dermal-epidermal adhesion in BP may also occur *via* complement-independent mechanisms (76, 81, 82), which are thought to be preponderant during early and non-blistering phases of BP, where non-fixing complement IgG4 subclasses are predominant (83–85). These mechanisms include (i) internalization of BP180 from the surface of keratinocytes after IgG binding to BP180 (86); (ii) direct release of cytokines, e.g., IL-6 and IL-8, from keratinocytes (87); (iii) induction of MC degranulation and eosinophil activation by IgE autoantibodies (88).

It is thus possible that both complement-dependent and complement-independent mechanisms work together in inducing and perpetuating BP inflammation and blistering (85).

## Cytokine regulation of non T/B immune cells in bullous pemphigoid

Loss of dermal-epidermal adhesion in BP is critically associated with skin infiltration of neutrophil and eosinophil granulocytes (89–91). In passive transfer models, pathogenicity of antibodies is significantly reduced in the absence of myeloid granulocytes (89, 92, 93). Moreover, BP models induced by genetic deletion of BP180 pathogenic domains are characterized by spontaneous infiltration of granulocytes (94). Granulocytes induce blistering by different mechanisms such as the release of proteases, e.g., MMP-9, reactive oxygen species (ROS) and either neutrophil or eosinophil extracellular traps (51, 92, 95–97) (Figure 1). In humans, neutrophils and eosinophils localize differently in BP skin, with the first predominating in the blister fluids and the second in the dermal skin (98). The blister fluid, as well as sera, of BP patients over-express several chemoattractant and pro-inflammatory molecules, including IL-1, IL-8, IL-10, IL-5, Tumor necrosis factor-alpha, IL-6, CCL17, CCL-1, galectin-9, periostin, CCL11, CCL26, thymic stromal lymphopoietin and eotaxins (99–107) (Table 1). Dermal-epidermal separation induced by eosinophils is mainly orchestrated by IL-5 and depends on adhesion and Fcγ receptor activation (108). Neutrophils and eosinophils also regulate different aspects of BP inflammation. As an example, NET release from neutrophils acts systemically by inducing B-cell differentiation into plasma cells *via* activation of the MAPK/p38 cascade (109). Eosinophils mediate specific IgE pathogenicity, release cytokines which enhance Th2 cell recruitment and stimulate peripheral nerve terminals, e.g., by releasing IL-31 (88, 110–112).

Cross talk between immune cells is likely to potentiate the effector functions of granulocytes. Accordingly, neutrophils in BP release significantly more ROS and MMP-9 when stimulated with monocyte supernatants *in vitro* (113). Recently, Granzyme B was shown to critically regulate monocyte-dependent neutrophil recruitment in BP, and its inhibition significantly ameliorated pemphigoid disease induced by immunization with anti-COL7 antibodies in mice (114).

Monocytes and neutrophils are also activated by CXCL10, whose levels are increased in early-relapsing patients and is produced by keratinocytes, fibroblasts, and infiltrating immune cells (115).

Tissue resident macrophages are increased in BP skin and are mainly polarized toward the M2 phenotype, which express CD163 and CD206 (116, 117). M2 macrophages produce large amounts of Th2-type cytokines and stimulate T-cell and eosinophil recruitment by releasing CCL18, CCL22, CCL24, and CCL2 (117, 118).

Furthermore, CD163+ M2 macrophages stimulated by LL37 *in vitro* produced CXCL10 and CCL20 as well CXCR5+, CXCL13+, which contribute to recruitment of TFH cells (43, 117).

Finally, BP skin is enriched in both basophils and MC. Basophils are implicated in BP-associated itch (119). The role of MCs is still matter of debate. Experimental evidence suggests that MC degranulation in mice with BP occur after different stimuli, including complement fractions and specific IgE antibodies (74). Finally, macrophage-mediated neutrophil infiltration depends on MC activation (120, 121) (Figure 1). However, while studies using KIT-dependent MC knock-out mice demonstrated that MC activation trigger BP (121), Kit-independent MC-deficient mice still develop the disease, without significant changes in immune cells infiltration. Collectively, these findings raise the hypothesis that MC activation could be a bystander effect of BP inflammation (122).

## Target therapies in bullous pemphigoid

Until now, several targeted therapies for BP have been developed, including (i) cell-depleting therapies; (ii) autoantibody-targeting therapies and (iii) single cytokine/molecule-directed therapies (Table 1).

Rituximab, a B-cell depleting therapy, is still not approved for BP, but often applied off-label to patients who fail conventional therapies. BP patients receiving one or more RTX cycles experience high rate of complete remission (CR) (approximately 75–92% of CR and 40% of CR off therapy), with significant drop of autoantibody titer (27–29, 123). Notably, RTX was shown to halve the 5-year mortality rate of BP in one study (124). Although RTX is mostly applied according to either the lymphoma protocol (375 mg/m2 weekly for 4 weeks) or the rheumatoid arthritis protocol (2 infusions of 1,000 mg 2 weeks apart), increasing number of studies addressed the efficacy of low (2 infusions of 500 mg 2 weeks apart) or ultra low (100 mg weekly for 4 weeks) doses of RTX in BP (125, 126).

Intravenous immunoglobulins showed pleiotropic anti-inflammatory effects (26), including increased autoantibody catabolism, and demonstrated meaningful positive effects in several cases and studies in BP both as monotherapy or combined with RTX (127–130). More recently, efgartigimod, a monoclonal antibody targeting the neonatal Fc receptor and thereby hastening the internalization and degradation of immunoglobulins, has entered clinical trials in BP after promising results in pemphigus and myasthenia gravis patients (131, 132). Several studies reported disease improvement with omalizumab, a monoclonal antibody targeting the IgE-specific Fc epsilon receptor III (133–137) (Table 1). Omalizumab offers a favorable safety profile, making it suitable for patients with contraindication to prolonged corticosteroid/immunosuppressive regimens (138, 139). Intriguingly, it showed efficacy also in patients without detectable serum anti-BP180/BP230 IgE. Responder patients show a decrease in IgE skin deposition, circulating IgG autoantibody levels and circulating basophils, which suggests immune-modulatory effects beyond IgE inhibition (137, 138, 140).

Over the recent years, complement activation has served as an attractive target in BP, owing to the established role in BP pathogenesis demonstrated in animal studies.

In one study blockage of C1s by the specific inhibitor, TNT003, successfully blocked the complement activating capacity of BP sera. Likewise, Gutjahr et al. (141) found that tinzaparin sodium inhibited autoantibody-induced complement activation in BP sera.

More recently, Sadik et al. (142) reported the results of a phase IIa non-randomized clinical trial of BP patients treated with nomacopan (NCT05061771), an inhibitor of leukotriene B4 and complement C5. Seven of the nine patients recruited demonstrated remarkable reduction of Bullous Pemphigoid Disease Area Index (BPDAI) and pruritus after approximately 1.5 months. No serious adverse events were reported (Table 1).

Since a first report in 2018 (143), dupilumab, an IL-4 alpha subunit receptor inhibitor has been used increasingly in BP. Dupilumab mainly acts by suppressing IL-4- and IL-13-producing CD4+ T cells (144). In a case series of 13 patients published in 2020, dupilumab demonstrated a satisfactory response in 92.3% (145). BP patients receiving dupilumab in combination with conventional therapies achieve faster clinical response with a reduction of the cumulative steroid dose compared to those treated with conventional therapies (146, 147). Interestingly, combination of omalizumab and dupilumab demonstrated efficacy in one case of recalcitrant BP (148). Finally, the drug holds promise as a treatment for special settings of patients, including highly recalcitrant, rituximab-resistant, or immune checkpoint inhibitor-induced BP (149–151). So far, attempts to block IL-5 (mepolizumab) failed to show an impact on BP activity (NCT01705795), while a study with benralizumab, a monoclonal antibody targeting the IL-5 receptor is currently ongoing (NCT04612790) (Table 1).

Finally, it will be intriguing to evaluate the efficacy of nemolizumab, a monoclonal antibody targeting IL-31, on disease activity and pruritus of BP.

## Concluding remarks

An intriguing aspect of the pathogenesis of BP is that antibody/antigen binding activates different pathways, which seem to act in parallel rather than as a single cascade. Hence, combining different target therapies will represent a feasible way to reduce the cumulative exposure of patients to systemic steroids. In a merely speculative manner, combination of rituximab and dupilumab might effectively target the T-B-cell cross-talk involved in the loss of tolerance against BP autoantigens; while, combination of anti-complement drugs and either neutrophil-or eosinophil-targeting therapies might be best suited to impair the effector phase of BP inflammation and pruritus. Indeed, with the number of available therapeutic options rapidly increasing, clinicians should focus on identifying comorbidities, clinical variables (e.g., bullous vs. non-bullous phenotypes and pruritus intensity), laboratory [e.g., neutrophil-rich vs. eosinophil-rich infiltrates at histopathology, or the intensity of complement deposition at direct immunofluorescence (DIF)] and serological findings (e.g., titer of IgG and IgE antibodies against BP180/BP230) or molecular factors (e.g., cytokine concentration) which may influence therapy-response and decision-making.

## Author contributions

RM contributed to the design, writing, and speculation. FS, DD, and CP contributed to the writing and speculation. EA contributed to the design and writing. GD contributed to the design, writing, and supervision. All authors contributed to the article and approved the submitted version.
